# Novel Ultrasound-Guided Bilateral Simultaneous Hydrodissection of the Cervical Sympathetic Chain and Vagus Nerves: A Detailed Technical Description With Anatomical Correlation

**DOI:** 10.7759/cureus.106250

**Published:** 2026-04-01

**Authors:** King-Hei Stanley Lam, Teinny Suryadi, Daniel Chiung-Jui Su, Anwar Suhaimi, Abdallah El-Sayed Allam, Yonghyun Yoon

**Affiliations:** 1 Pain Management, Faculty of Medicine, The Chinese University of Hong Kong, New Territories, HKG; 2 Pain Management, Faculty of Medicine, The University of Hong Kong, Hong Kong, HKG; 3 Musculoskeletal Medicine, The Board of Clinical Research, The Hong Kong Institute of Musculoskeletal Medicine, Kowloon, HKG; 4 Physical Medicine and Rehabilitation, Medistra Hospital, Jakarta, IDN; 5 Physical Medicine and Rehabilitation, Synergy Clinic, Jakarta, IDN; 6 Physical Medicine and Rehabilitation, Hermina Hospital Podomoro, Jakarta, IDN; 7 Physical Medicine and Rehabilitation, Chi Mei Medical Center, Tainan, TWN; 8 Rehabilitation Medicine, Universiti Malaya Medical Centre, Universiti Malaya, Kuala Lumpur, MYS; 9 Physical Medicine and Rehabilitation, Morphological Madrid Research Center (MoMaRC), UltraDissection Spain EchoTraining School, Madrid, ESP; 10 Physical Medicine, Rheumatology, and Rehabilitation, Tanta University Hospitals and Faculty of Medicine, Tanta University, Tanta, EGY; 11 Orthopedics, The Board of Clinical Research, The International Association of Musculoskeletal Medicine, Kowloon, HKG; 12 Orthopedics, International Academy of Regenerative Medicine, Incheon, KOR; 13 Orthopedics, MSKUS, San Diego, USA; 14 Orthopedic Surgery, Hallym University Kangnam Sacred Heart Hospital, Seoul, KOR; 15 Orthopedic Surgery, Incheon Terminal Orthopedic Surgery Clinic, Incheon, KOR

**Keywords:** 5% dextrose in water without local anesthetic, autonomic nervous system, carotid sheath, cervical sympathetic chain, long covid symptoms, longus colli, prevertebral fascia, stellate ganglion, ultrasound-guided hydrodissection, vagus nerve

## Abstract

The vagus nerve and cervical sympathetic chain regulate autonomic function and are implicated in chronic pain and post-viral autonomic syndromes. Hydrodissection with 5% dextrose is established for peripheral nerves, but simultaneous ultrasound-guided hydrodissection of both cervical autonomic nerves has not been described. We describe a reproducible ultrasound-guided technique for bilateral simultaneous hydrodissection of the cervical sympathetic chain and vagus nerves using 5% dextrose without local anesthetic. The procedure uses a high-frequency linear transducer at C6-C7 and an in-plane lateral 25-gauge needle approach, performing the deeper sympathetic chain hydrodissection first (prevertebral fascia superficial to the longus colli), followed by vagus nerve hydrodissection within the carotid sheath. Injection is performed slowly (12-18 minutes per side) to permit full delivery and patient comfort. We report procedural outcomes in 10 patients (30 sessions) treated for post-COVID-related autonomic dysfunction.

All procedures were completed successfully. Total injectate was 60 mL of 5% dextrose per side (approximately 30 mL for the sympathetic chain and 30 mL for the vagus nerve). The deep-to-superficial sequence preserved sonographic visualization and avoided air-bubble artifact. No procedural complications occurred (including bradycardia, hypotension, voice changes, dysphagia, hematoma, or infection). At a minimum 12-month follow-up, patients maintained clinical improvement without additional interventions. Ultrasound-guided bilateral simultaneous hydrodissection of the cervical sympathetic chain and vagus nerves with 5% dextrose is technically feasible and well tolerated. A deep-to-superficial injection sequence and very slow administration optimize visualization and delivery. Detailed clinical outcomes are presented separately.

## Introduction

The vagus nerve (cranial nerve X) and the cervical sympathetic chain are essential components of the autonomic nervous system, playing critical roles in regulating cardiac activity, respiratory function, gastrointestinal processes, and immune responses through the cholinergic anti-inflammatory pathway [1,2]. Dysfunction of these nerves has been implicated in various chronic pain conditions, autonomic disorders, and post-viral syndromes, including post-COVID syndrome [3,4]. However, chronic pain conditions often involve complex mechanisms beyond autonomic dysfunction alone, including nociplastic pain mechanisms that may not be adequately addressed by traditional sympathetic blocks alone [5].

The cervical sympathetic chain includes the stellate ganglion, which is formed by the fusion of the inferior cervical and first thoracic ganglia. It plays a critical role in sympathetic innervation to the upper extremities, head, neck, and heart [6]. The stellate ganglion (cervicothoracic ganglion) lies anterolateral to the C7 and T1 vertebral bodies, receives input from the paravertebral sympathetic chain, and has been a traditional target for sympathetic blocks in conditions such as complex regional pain syndrome, post-herpetic neuralgia, chronic pain of the head and neck, and post-traumatic stress disorder [7-14]. Traditionally, local anesthetic solutions have been injected as blocks for these conditions [15-17].

Nerve hydrodissection with 5% dextrose in water (D5W) has emerged as a safe and effective treatment for various nerve entrapment and dysfunction syndromes [18-20]. Unlike corticosteroid or local anesthetic injections, D5W mechanically releases nerves from surrounding fascial restrictions while exerting beneficial metabolic effects on inflamed neural tissue, including reductions in reactive oxygen species production and apoptosis [21,22]. Meta-analyses have confirmed the efficacy of D5W hydrodissection for carpal tunnel syndrome, with effects superior to those of saline or corticosteroids [23-25].

Understanding the normal ultrasound morphology of the cervical vagus nerve is essential for successful hydrodissection. High-resolution ultrasound studies have provided valuable reference data for clinical practice. Drakonaki et al. examined 657 cervical vagus nerves in 330 individuals without neurological disease and established that the vagus nerve typically appears as a monofascicular or oligofascicular structure with a honeycomb appearance, containing between one and six discrete fascicles [26]. The right vagus nerve is typically larger than the left and contains more fascicles, a normal asymmetry that should be anticipated during bilateral procedures. The cross-sectional area correlates positively with body mass index but shows no significant association with age or sex. These normative data provide important reference values for identifying pathological changes and guide expectations during hydrodissection procedures [26].

Ultrasound-guided hydrodissection of the cervical sympathetic chain using D5W has been previously reported for the treatment of chronic upper trunk pain [27]. The technique involves approaching the prevertebral fascia superficial to the longus colli muscle, with the needle tip positioned just ventral to the anterior tubercle of C6, and using hydrodissection to allow fluid to track caudally to reach the stellate ganglion at the C7 and T1 level [27]. Additionally, hydrodissection of the cervical plexus with D5W has shown promise in treating post-traumatic stress disorder, suggesting broader applications for cervical nerve hydrodissection in conditions involving autonomic dysregulation [28]. However, to our knowledge, simultaneous hydrodissection of both the cervical sympathetic chain, with caudal tracking to the stellate ganglion, and the vagus nerves has not been previously described in detail.

The carotid sheath, located at the C6-C7 vertebral level, provides a unique anatomical opportunity to access both the vagus nerve and the cervical sympathetic chain in a single procedural approach [29,30]. The vagus nerve lies within the carotid sheath between the common carotid artery and internal jugular vein, while the cervical sympathetic chain runs posterior to the carotid sheath, embedded within the prevertebral fascia overlying the longus colli muscle [31]. The stellate ganglion is located more caudally at the C7 level, anterolateral to the C7 vertebral body, and can be reached by fluid tracking within the prevertebral fascia [6,27].

The ultrasonographic appearance of the vagus nerve, as described by Drakonaki et al., is consistently observed at the C6-C7 level as a hyperechoic oval or triangular structure containing hypoechoic fascicles, typically in a monofascicular or oligofascicular honeycomb pattern [26]. The right vagus nerve is typically larger with more fascicles than the left, a finding that should be anticipated during bilateral procedures. The cross-sectional area varies with body mass index, and this variability should be considered when assessing for pathological enlargement or compression. Recognition of these normal morphological patterns facilitates accurate nerve identification and helps distinguish the vagus nerve from the smaller cervical sympathetic chain located posterior to the carotid sheath.

A critical technical consideration when targeting multiple structures in the same region is the sequence of injection. Injecting superficial structures first can introduce air bubbles or fluid that create shadowing and distortion, thereby obscuring visualization of deeper targets [32]. Therefore, when performing simultaneous hydrodissection of the cervical sympathetic chain, which lies deep, and the vagus nerve, which is relatively more superficial within the carotid sheath, it is essential to hydrodissect the deeper cervical sympathetic chain first, followed by the more superficial vagus nerve. This deep-to-superficial sequence ensures clear, uninterrupted visualization of both targets throughout the procedure.

The use of D5W without local anesthetic is critical for bilateral same-session treatment, as local anesthetics would risk bilateral vagal blockade with potentially serious consequences, including bradycardia, hypotension, and respiratory compromise. D5W, in contrast, appears to restore normal neural function rather than blocking it, as evidenced by the absence of adverse effects in peripheral nerve hydrodissection [18-20,32].

Another critical technical factor is the rate of injection. Very slow injection over 12-18 min per side allows for the delivery of the full 60 mL volume (30 mL for the sympathetic chain and 30 mL for the vagus nerve) with excellent patient tolerance. Rapid injection can cause patient discomfort, tissue distension, and suboptimal fluid distribution. The slow injection technique allows the hydrodissecting fluid to gradually separate tissue planes, minimizing discomfort and giving the patient time to adapt to the procedure.

This technical report provides a detailed description of a novel approach to ultrasound-guided bilateral simultaneous hydrodissection of the cervical sympathetic chain, with caudal tracking to the stellate ganglion and the vagus nerves, using 5% dextrose in water without local anesthetic. This report includes a discussion of sonoanatomy, needle guidance, the critical deep-to-superficial injection sequence, the importance of very slow injection for patient tolerance, the hydrodissection technique, and safety considerations. We also present procedural outcomes from 10 patients with post-COVID syndrome who underwent this procedure, demonstrating its feasibility and safety. Detailed clinical outcomes for these patients will be reported in a separate manuscript.

## Technical report

Technical description

Anatomical Considerations - Critical Neurovascular Structures to Avoid

Prior to undertaking this procedure, a thorough understanding of the dangerous structures in the cervical region is essential to ensure patient safety and prevent iatrogenic injury. The vertebral artery and vein represent the most critical vascular structures to avoid when approaching the cervical sympathetic chain, as inadvertent intravascular injection can lead to catastrophic complications, including hematoma, seizure, or stroke. These vessels are typically located medial to the C7 nerve root at the level of the C7 posterior tubercle, and mandatory Doppler confirmation is required to distinguish them from neural structures before proceeding, as their gray-scale appearance may closely mimic that of a nerve root.

The cervical nerve roots, particularly C5, C6, and C7, are critical neural structures that should be avoided during needle advancement. These nerve roots exit through the neural foramina and traverse the region between the scalenus anterior and scalenus medius muscles before forming the brachial plexus. Inadvertent needle contact or injection into a nerve root may precipitate acute radicular pain, paresthesias, or, in rare cases, persistent nerve injury. Careful continuous visualization of the needle tip throughout the procedure, combined with hydrodissection ahead of the needle, minimizes the risk of unintended neural contact.

The phrenic nerve is another critical structure that requires particular attention during this procedure. Originating from the C3, C4, and C5 nerve roots, the phrenic nerve courses obliquely across the anterior surface of the scalenus anterior muscle, positioned directly in the path of needle advancement toward the prevertebral fascia. Injury to the phrenic nerve may result in diaphragmatic dysfunction or hemidiaphragmatic paralysis, which can present clinically as dyspnea, orthopnea, or reduced exercise tolerance. The risk of phrenic nerve injury is minimized by using hydrodissection to gently separate tissues ahead of the needle, avoiding local anesthetic in the injectate, and maintaining the needle in a plane deep to the prevertebral fascia by gently hydrodissecting through the scalenus anterior muscle to avoid injury to the phrenic nerve.

The recurrent laryngeal nerve, a branch of the vagus nerve, courses in the tracheoesophageal groove and is vulnerable to injury if the needle is advanced too medially. Injury to this nerve may result in temporary or persistent hoarseness due to ipsilateral vocal cord paresis or paralysis. The risk of recurrent laryngeal nerve injury is minimized by gently using hydrodissection to separate tissues ahead of the needle, avoiding local anesthetic in the injectate, maintaining the needle tip in view at all times before advancing the needle, and avoiding the needle tip from touching the tracheoesophageal groove.

The common carotid artery and internal jugular vein are major vascular structures that warrant particular emphasis during the vagus nerve hydrodissection component of this procedure. These vessels lie within the carotid sheath in close proximity to the vagus nerve, with the nerve typically situated between and slightly posterior to them. During vagus nerve hydrodissection, the needle is intentionally directed into the carotid sheath, placing these vessels at direct risk. Inadvertent puncture of the common carotid artery may result in hematoma formation, vessel dissection, or, in rare cases, thromboembolic complications, while puncture of the internal jugular vein carries risks of hematoma, venous thrombosis, or air embolism. Furthermore, intravascular injection of the hydrodissection solution, though less hazardous than injection of local anesthetic, may still result in unintended systemic distribution and theoretical risks of vascular injury. To mitigate these risks, the needle tip should be continuously visualized during entry into and manipulation within the carotid sheath. Negative aspiration should be performed before injection, and any unexpected resistance or lack of fluid spread should prompt immediate reassessment of the needle tip position. The use of color Doppler or power Doppler to confirm the location of these vessels relative to the needle trajectory is strongly recommended before and during the procedure.

Recognition of these negative landmarks is as critical as identification of the target structures for safe procedure performance. The use of color Doppler or power Doppler to confirm vascular structures is not merely recommended but is mandatory, as it provides the definitive distinction between the vertebral vessels and adjacent neural elements, thereby preventing what would otherwise be a potentially catastrophic complication.

Vagus nerve anatomy: At the C6-C7 vertebral level, the vagus nerve is consistently located within the carotid sheath, between and slightly posterior to the common carotid artery and internal jugular vein [31]. High-resolution ultrasound enables detailed visualization of the nerve's internal architecture. Based on the normative data from Drakonaki et al., the cervical vagus nerve typically appears as a monofascicular or oligofascicular structure with a honeycomb appearance, containing one to six discrete fascicles [26]. The right vagus nerve is normally larger with more fascicles than the left, and its cross-sectional area correlates positively with body mass index. These morphological features aid in accurate nerve identification and differentiation from the smaller cervical sympathetic chain located posterior to the carotid sheath. The recurrent laryngeal nerve branches from the vagus inferior to this level, rendering the C6-C7 level safe for intervention.

Cervical sympathetic chain and stellate ganglion anatomy: The cervical sympathetic chain runs posterior to the carotid sheath, embedded within the prevertebral fascia overlying the longus colli muscle [6,27]. The stellate ganglion is formed by the fusion of the inferior cervical and first thoracic ganglia and lies anterolateral to the C7 vertebral body [5]. It receives input from the paravertebral sympathetic chain and provides sympathetic efferents to the upper extremities, head, neck, and heart [7-9].

The longus colli muscle serves as a key landmark for accessing the sympathetic chain [27,33]. The anterior tubercle of the C6 vertebral body, known as the Chassaignac tubercle or carotid tubercle, is an important landmark located superior to the stellate ganglion [26]. The C7 vertebra does not possess an anterior tubercle, while the anterior tubercle of C5 is less prominent. Therefore, the anterior tubercle of C6 can be easily identified and serves as a reliable reference point [27].

A cadaveric study utilizing dye injection with subsequent clinical validation has demonstrated adequate spread of solution to the stellate ganglion using a technique in which the needle tip is positioned deep to the prevertebral fascia to avoid spread along the carotid sheath and superficial to the fascia investing the longus colli to prevent injection into the muscle substance [33].

Rationale for Deep-to-Superficial Injection Sequence

When performing hydrodissection of multiple targets within the same anatomical region, the injection sequence is critical for maintaining optimal sonographic visualization [32]. If the more superficial structure, namely the vagus nerve within the carotid sheath, is injected first, several problems may occur. First, even minimal introduction of air bubbles during the superficial injection creates hyperechoic shadowing that obscures deeper structures [32]. Second, accumulation of injectate in the superficial plane distorts the tissue architecture and compresses potential spaces, making deeper needle advancement more difficult. Third, the normal relationship among the carotid artery, the internal jugular vein, and the surrounding fascia may be altered, compromising identification of the deeper sympathetic chain.

Therefore, the cervical sympathetic chain, as the deep structure, should be hydrodissected first, followed by the vagus nerve as the more superficial structure. This sequence ensures that the deeper target is accessed through undisturbed anatomy with clear visualization, and any subsequent injection into the superficial plane does not compromise the already completed deep hydrodissection.

Patient Positioning

The patient is positioned supine on the procedure table, with a rolled towel placed beneath the neck to provide slight extension, and another thin pillow or rolled towel placed beneath the ipsilateral shoulder to facilitate slight rotation of the head 15° contralateral to the point of needle entry [27]. This positioning optimizes exposure of the anterior neck region while maintaining patient comfort and airway patency.

Continuous pulse oximetry, heart rate monitoring, and non-invasive blood pressure measurements are established prior to procedure initiation. Intravenous access is not routinely required but should be available. Resuscitation equipment, including atropine and airway management supplies, should be immediately accessible.

Ultrasound Equipment and Transducer Placement

A high-frequency linear ultrasound transducer (6-18 MHz) is used for all procedures. The ultrasound machine is positioned on the contralateral side of the neck to optimize ergonomics and maintain an unobstructed view of the screen during needle advancement.

The transducer is placed transversely on the anterior neck at the level of the C6-C7 vertebrae, identified by the presence of the carotid tubercle (Chassaignac tubercle) on the transverse process of C6 and the characteristic appearance of the vertebral bodies. At this level, the following structures are identified in transverse view from medial to lateral: the trachea appears as a midline structure with hyperechoic air artifact; the esophagus on the left side or the thyroid gland on the right side is located posterolateral to the trachea; the longus colli muscle overlies the vertebral body, appearing as a hypoechoic structure with linear hyperechoic striations; the common carotid artery presents as a round, anechoic, pulsatile structure lateral to the thyroid; the internal jugular vein appears as a larger, anechoic, compressible structure lateral and slightly anterior to the common carotid artery; the vagus nerve is located between and slightly posterior to the common carotid artery and internal jugular vein within the carotid sheath, appearing as a small hyperechoic oval or triangular structure; and the cervical sympathetic chain is located posterior to the carotid sheath, embedded within the prevertebral fascia overlying the longus colli muscle, appearing as a small hypoechoic oval or triangular structure distinct from the larger vagus nerve.

Color Doppler or power Doppler should be used to confirm vascular structures, as this is a critical safety measure. Without a Doppler signal, the vertebral artery and vein, which are typically located medial to the C7 nerve root at the level of the C7 posterior tubercle, may be mistaken for neural structures, potentially leading to inadvertent intravascular injection with catastrophic consequences, including hematoma, seizure, or stroke. The anterior tubercle of C6 and the C6 nerve root should also be identified as additional landmarks for accessing the sympathetic chain [26].

Needle Selection and Preparation

A 25-gauge or 27-gauge 2-inch (50 mm) hypodermic needle is used for the procedure. Smaller gauge needles, such as 27G, may provide greater patient comfort but require slower injection due to higher flow resistance. The needle is connected to a 10 mL Luer-lock syringe filled with 5% dextrose in water (D5W). Multiple syringes are prepared to deliver the total volume of 60 mL per side.

Injectate

The injectate used exclusively is D5W without the addition of any local anesthetic, corticosteroid, or other adjuvant. D5W is prepared as a standard commercial solution containing 5 g of dextrose per 100 mL in sterile water. No additives are used. The total volume per side is 60 mL, comprising 30 mL for cervical sympathetic chain hydrodissection and 30 mL for vagus nerve hydrodissection.

Critical Importance of Slow Injection Rate

Injection rate is a critical technical factor for patient tolerance and optimal fluid distribution. Very slow injection over 12-18 min per side allows delivery of the full 60 mL volume with excellent patient tolerance. Rapid injection can cause several problems, including patient discomfort from acute distension of tissue planes that may lead to anxiety and patient movement, suboptimal fluid distribution from turbulent flow that reduces the efficacy of hydrodissection, and increased risk of tissue trauma as the hydrodissection technique relies on fluid gently separating tissue planes ahead of the needle tip rather than overcoming tissue resistance too quickly. The slow injection technique ensures that the hydrodissecting fluid gradually separates tissue planes, minimizes discomfort, and allows the patient to adapt to the procedure. In the rare event that a patient experiences discomfort during the injection, the procedure is paused briefly and resumed after a short rest.

Needle Guidance and Hydrodissection Technique

Notably, the cervical sympathetic chain, as the deep structure, must be hydrodissected first, followed by the vagus nerve, which is more superficial within the carotid sheath. This deep-to-superficial sequence ensures optimal sonographic visualization throughout the procedure.

Step 1 (skin entry and superficial hydrodissection): Under continuous ultrasound visualization and after skin numbing, the needle is inserted from the lateral aspect of the neck using an in-plane technique. The needle tip is continuously visualized throughout the procedure. Hydrodissection is performed from the point of skin entry using D5W, creating a fluid-filled tract ahead of the needle tip that gently separates soft tissues and minimizes trauma [18,34,35]. Slow, steady injection of D5W creates an anechoic halo that precedes the needle tip, allowing visualization of the path and the displacement of small vessels and nerves that are too small to be individually identified. This hydrodissection technique, ahead of the needle, is fundamental to safety. The injection rate is maintained very slowly throughout the procedure, typically requiring 12-18 min per side to deliver the full 60 mL volume.

Step 2 (advancing through platysma and investing fascia): The needle is advanced through the subcutaneous tissue and the platysma muscle, which is identified as a thin hypoechoic layer just beneath the subcutaneous fat. Continuous hydrodissection facilitates passage through the investing fascia of the sternocleidomastoid muscle. At this stage, the needle is directed toward the deeper prevertebral space, posterior to the carotid sheath, to access the cervical sympathetic chain first.

Step 3 (identification of landmarks for sympathetic chain access): The longus colli muscle, vertebral artery and vein, C7 nerve root, and the posterior tubercle of C7 are the key landmarks identified [26]. The needle is advanced toward the prevertebral fascia superficial to the longus colli, passing through the scalenus anterior muscle to reach the junction between the scalenus anterior and longus colli [27].

**Figure 1:**
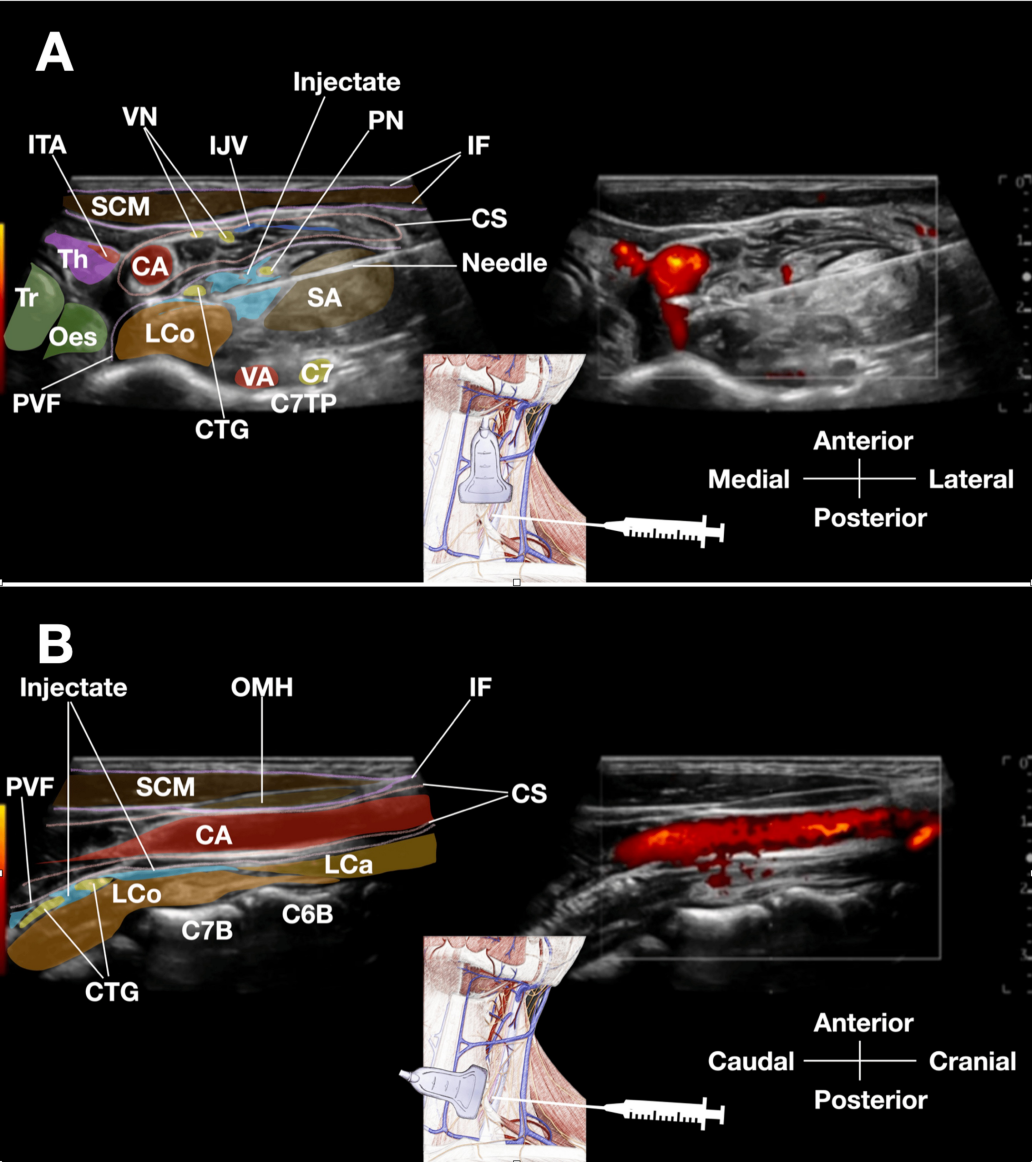
Hydrodissection of the cervical sympathetic chain with caudal tracking to the cervicothoracic ganglion. (A) Transverse ultrasound view at the C7 level during cervical sympathetic chain hydrodissection. The color-labeled sonogram demonstrates key anatomical structures during hydrodissection of the cervical sympathetic chain. The needle is positioned within the prevertebral fascia superficial to the longus colli muscle, with the bevel turned downward to direct injectate flow. The injectate (anechoic fluid, represented by light blue shading) separates the prevertebral fascia from the longus colli, surrounding the cervical sympathetic chain. The carotid sheath contents (common CA, IJV, VN) remain undisturbed at this stage, illustrating the advantage of the deep-to-superficial injection sequence. (B) Sagittal ultrasound view confirming caudal fluid tracking to the cervicothoracic ganglion. The color-labeled sagittal sonogram, obtained with the transducer rotated 90°, demonstrates caudal tracking of injectate within the prevertebral fascial plane. The needle tip is positioned at the level of the C7 vertebral body. The injected 5% dextrose in water (anechoic fluid, represented by blue shading) tracks caudally along the prevertebral fascia superficial to the longus colli muscle to reach the cervicothoracic ganglion at the C7 and T1 vertebral levels, confirming successful hydrodissection of the entire cervical sympathetic chain with extension to the stellate ganglion region. C6B: C6 vertebral body; C7: C7 nerve root; C7B: C7 vertebral body; C7PT: C7 posterior tubercle; CA: common carotid artery; CS: carotid sheath; CTG: cervicothoracic ganglion; IF: investing fascia of sternocleidomastoid; IJV: internal jugular vein; LCa: longus capitis; LCo: longus colli; Oes: esophagus; OMH: omohyoid; PVF: prevertebral fascia; SA: scalenus anterior; SCM: sternocleidomastoid; SM: scalenus medius; SP: scalenus posterior; Th: thyroid; Tr: trachea; VA: vertebral artery; VN: vagus nerve; VV: vertebral vein The color shading and labeling were created in Keynote by Professor King-Hei Stanley Lam.

Step 4 (cervical sympathetic chain hydrodissection {30 mL}): Once the needle tip hydrodissects and reaches the layer deep to the prevertebral fascia and superficial to the longus colli, the bevel of the needle is turned downward so that the injectate pushes the soft tissues in front of and beneath the needle [27]. This orientation is critical for directing flow between the prevertebral fascia and the longus colli muscle. Figure 1, panel A, provides a detailed color-labeled transverse sonogram demonstrating this step, showing the needle positioned within the prevertebral fascia superficial to the longus colli, with the injectate surrounding the cervical sympathetic chain (Video 1).

**Video 1:** Ultrasound-guided simultaneous hydrodissection of the cervical sympathetic chain and vagus nerve. The full procedure for simultaneous hydrodissection of the left cervical sympathetic chain and left vagus nerve using 5% dextrose in water without local anesthetic as the injectate is shown. Color-labeled still images are incorporated to illustrate the detailed sonoanatomy at each critical step. Throughout the procedure, critical neurovascular structures are carefully identified and avoided, including the vertebral artery and vein (confirmed with color Doppler to distinguish them from the C7 nerve root), the phrenic nerve on the anterior scalene muscle, the recurrent laryngeal nerve in the tracheoesophageal groove, and the common carotid artery and internal jugular vein within the carotid sheath. The deep-to-superficial injection sequence (sympathetic chain first, vagus nerve second) and the very slow injection rate are maintained to optimize visualization, patient tolerance, and safety. C5: C5 nerve root; C6: C6 nerve root; C7: C7 nerve root; C6B: C6 vertebral body; C7B: C7 vertebral body; C7PT: C7 posterior tubercle; CA: common carotid artery; CS: carotid sheath; CTG: cervicothoracic ganglion; IF: investing fascia of sternocleidomastoid; IJV: internal jugular vein; LCa: longus capitis; LCo: longus colli; Oes: esophagus; OMH: omohyoid; PVF: prevertebral fascia; SA: scalenus anterior; SCM: sternocleidomastoid; SM: scalenus medius; SP: scalenus posterior; Th: thyroid; Tr: trachea; VA: vertebral artery; VN: vagus nerve; VV: vertebral vein The color shading and labeling in the still images embedded in the video were created in Keynote by Professor King-Hei Stanley Lam.

The objective is to use the force of the injectate to open a potential space between the prevertebral fascia and the longus colli [27]. Very slow, steady injection of D5W creates hydrodissection within this plane. The full 30 mL for sympathetic chain hydrodissection is delivered over approximately 6-9 min, depending on the patient's tolerance and the extent of adhesions encountered.

To confirm caudal tracking, the probe can be turned 90° to obtain a sagittal image through the needle tip, which is positioned on top of the C7 vertebral body, distal to the insertion of the longus capitis [27]. Upon continued hydrodissection, the fluid will be observed tracking caudally within the prevertebral fascia to reach the stellate ganglion at the C7 and T1 level [26]. Figure 1, panel B, presents a sagittal sonogram confirming this caudal fluid tracking to the cervicothoracic ganglion (Video 1).

The cervical sympathetic chain, embedded within the prevertebral fascia, becomes surrounded by D5W as the fluid dissects this plane. The typical volume required for sympathetic chain hydrodissection with caudal tracking to the stellate ganglion is 30 mL.

An important advantage of the deep-first sequence is that at this point, no fluid has been injected into the more superficial carotid sheath. Consequently, the anatomical relationships between the carotid artery, internal jugular vein, and vagus nerve remain undisturbed, providing clear visualization for the subsequent vagus nerve hydrodissection.

Step 5 (withdrawal and redirection for vagus nerve access): After completing cervical sympathetic chain hydrodissection with the full 30 mL, the needle is withdrawn partially until the tip is positioned just lateral to the carotid sheath. The absence of air bubbles or fluid artifact in the superficial planes ensures continued clear visualization of the carotid sheath contents.

Step 6 (vagus nerve hydrodissection {30 mL}): The needle is redirected medially to enter the carotid sheath by hydrodissection. Entry into the carotid sheath is confirmed by visualization of the needle tip within the sheath, by the characteristic compression of the internal jugular vein by the anechoic fluid and its separation from the common carotid artery as injectate accumulates, and by distension of the sheath with anechoic fluid.

**Figure 2:**
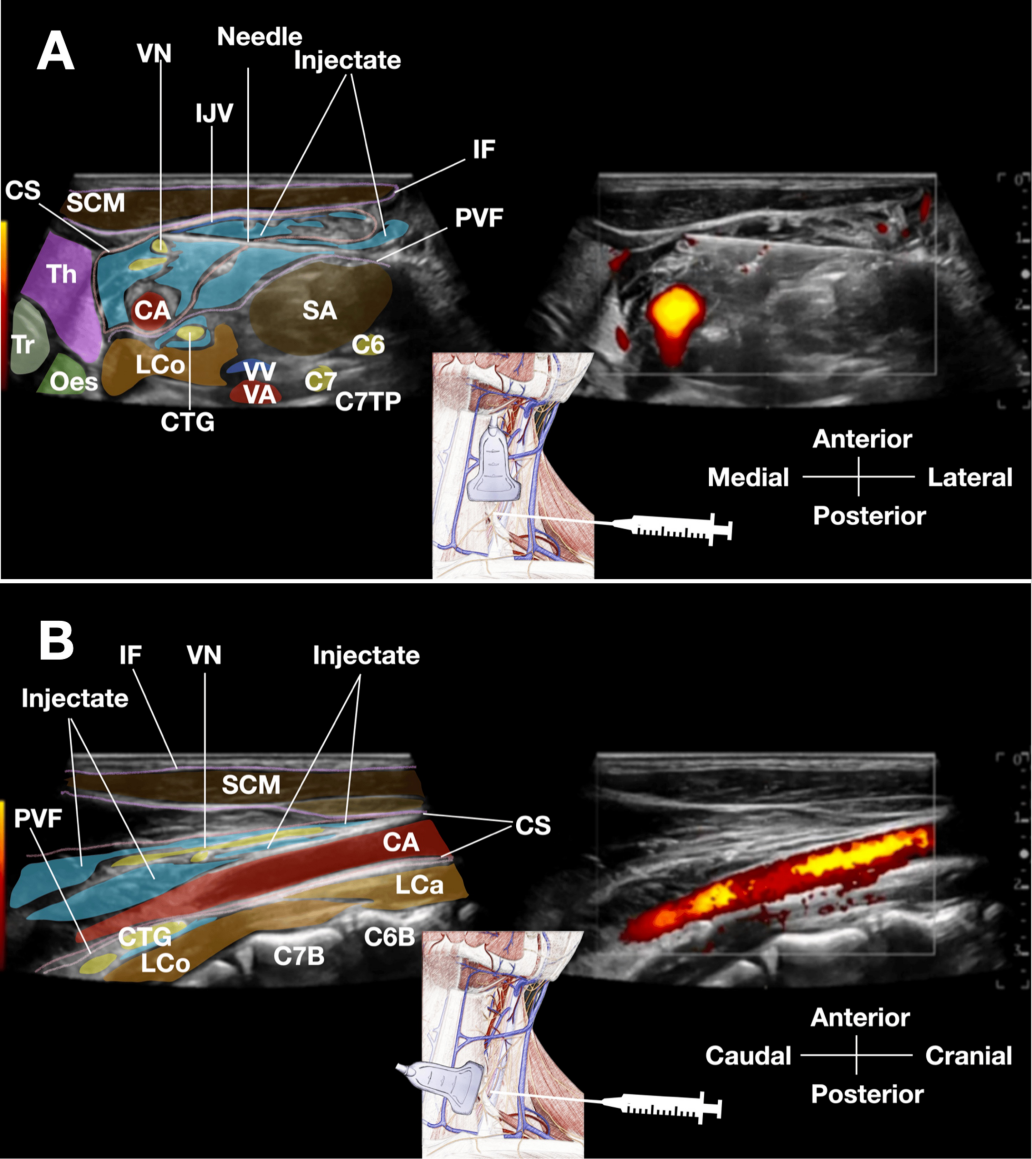
Hydrodissection of the vagus nerve within the carotid sheath. (A) Transverse ultrasound view at the C7 level during vagus nerve hydrodissection. The color-labeled sonogram demonstrates hydrodissection of the VN within the carotid sheath after sympathetic chain hydrodissection. The needle has been redirected to enter the CS. Slow 5% dextrose injection in water (anechoic fluid, colored in light blue) creates a 360° fluid halo completely surrounding the vagus nerve, separating it from the posteromedial wall of the common CA and surrounding fascial attachments. Of note, the characteristic compression and separation of the IJV from the carotid artery occurs as the injectate accumulates within the sheath. The previously hydrodissected CSC is visible posterior to the prevertebral fascia. (B) A sagittal ultrasound view of the vagus nerve within the carotid sheath. The color-labeled sagittal sonogram demonstrates the longitudinal view of the VN during hydrodissection. The transducer has been rotated 90° to obtain this sagittal plane through the carotid sheath. The needle is positioned within the sheath (not shown), and the injected 5% dextrose in water (bluish coloration) surrounds the vagus nerve along its longitudinal axis, confirming complete perineural hydrodissection. The vagus nerve is visualized as a hypoechoic tubular structure with hyperechoic borders, now separated from the surrounding fascial planes by the injectate. The common CA is seen in its longitudinal orientation. C7: C7 nerve root; C7B: C7 vertebral body; C7PT: C7 posterior tubercle; CA: common carotid artery; CSC: cervical sympathetic chain; CS: carotid sheath; IJV: internal jugular vein; IF: investing fascia of sternocleidomastoid; LCa: longus capitis; LCo: longus colli; Oes: esophagus; OMH: omohyoid; SA: scalenus anterior; SCM: sternocleidomastoid; SM: scalenus medius; SP: scalenus posterior; Th: thyroid; Tr: trachea; VA: vertebral artery; VN: vagus nerve; VV: vertebral vein The color shading and labeling were created in Keynote by Professor King-Hei Stanley Lam.

Within the carotid sheath, the needle tip is slowly hydrodissected to be positioned adjacent to the vagus nerve. Very slow injection of D5W is performed to separate the nerve from the common carotid artery. The vagus nerve, initially adherent to the artery, gradually lifts away as fluid surrounds it, creating a complete fluid halo (Figure 2, panels A and B). The full 30 mL for vagus nerve hydrodissection is delivered over approximately 6-9 min, again using very slow injection to ensure patient comfort and optimal fluid distribution.

The goal is to achieve 360° separation of the vagus nerve from surrounding soft tissues within the carotid sheath. The typical volume required for complete vagal hydrodissection is 30 mL (Video 1).

Step 7 (contralateral procedure): The procedure is then repeated on the contralateral side during the same session. The patient's head is repositioned with slight extension and turned away from the new treatment side. Fresh sterile preparation and a new needle are used for the contralateral side. The same deep-to-superficial sequence is maintained, with the cervical sympathetic chain hydrodissected first using 30 mL, followed by the vagus nerve using 30 mL, both delivered very slowly over 12-18 min total for that side.

Bilateral simultaneous hydrodissection is performed in all patients during each treatment session, enabled by the absence of local anesthetic, which eliminates concern for bilateral sympathetic and vagal blockade. The total procedure time for both sides ranges from 24 to 36 min, with a slow injection rate to ensure patient comfort throughout.

Management of Patient Tolerance

With the very slow injection technique described above, all patients in our series tolerated the full 60 mL per side. However, practitioners should be prepared to manage the rare situation where a patient experiences discomfort. In such cases, injection should be paused immediately if the patient reports significant discomfort, followed by a brief rest period of 30-60 s. Injection should then resume at an even slower rate when the patient is comfortable. If discomfort persists, verification that the needle tip position is correct and that hydrodissection is occurring in the intended plane should be performed. The patient should be reassured that slow injection is safe and that pausing is normal. In our experience, the slow injection technique pre-empts most discomfort, and all patients completed the full 60 mL per side without requiring volume reduction.

Treatment Protocol

Based on our clinical experience with peripheral and cervical nerve hydrodissection, we administer three treatment sessions at monthly intervals (baseline, one month, and two months) [26]. This interval allows assessment of response and cumulative benefit from serial treatments.

Intra-procedural Monitoring

Throughout each procedure, patients remain awake and responsive. Continuous monitoring includes heart rate and pulse oximetry, clinical assessment of voice quality (by asking the patient to speak), clinical assessment of swallowing function (by asking the patient to swallow), and blood pressure measurement before and after the procedure.

The operator maintains verbal communication with the patient between syringe exchanges, instructing the patient to report any discomfort, pain, electric shock sensations, or voice changes immediately. The very slow injection rate facilitates this communication.

Following completion of the bilateral procedure, patients are monitored for at least 30 min for any delayed adverse effects, including bradycardia (heart rate <60 bpm), hypotension (systolic blood pressure <90 mmHg), voice changes or hoarseness, dysphagia or swallowing difficulties, hematoma formation, and vasovagal reactions.

Results

Patient Cohort

The procedure was performed in 10 patients, comprising six females and four males, with a mean age of 46.2 ± 8.4 years (range = 34-59 years). All patients presented with post-COVID syndrome, with symptom duration of 12-36 months (mean = 21.4 ± 7.8 months). Each patient exhibited chronic multisite neuromuscular pain, autonomic dysfunction, and multi-system symptoms affecting the cardiovascular, respiratory, gastrointestinal, cognitive, and sleep domains. All patients had previously been diagnosed with fibromyalgia and had failed both pharmacological therapy and physiotherapy. They also reported only transient benefit from antidepressant medications, with improvement in sleep and some dysfunctions lasting no more than one to two months.

Procedural Outcomes

The procedure was successfully performed in all 10 patients across 30 treatment sessions (three sessions per patient at monthly intervals). The mean procedure time was 12-18 min per side, with the very slow injection technique ensuring excellent patient tolerance throughout. The total procedure time for bilateral treatment ranged from 24 to 36 min.

The mean injectate volume was 60 mL of D5W per side, comprising 30 mL for cervical sympathetic chain hydrodissection with caudal tracking to the cervicothoracic ganglion and 30 mL for vagus nerve hydrodissection within the carotid sheath. All 10 patients tolerated the full 60 mL per side with the slow injection technique. In a few instances, brief 30 s pauses were required when patients reported mild discomfort, but after resuming at a slower rate, all patients successfully received the full volume. No patient required a reduction of the target volume.

Pre-procedural ultrasound assessment of the vagus nerve in all 10 patients demonstrated morphology consistent with the normative data reported by Drakonaki et al. [26]. The right vagus nerve exhibited a typical honeycomb or monofascicular pattern and was slightly larger in cross-sectional area than its left counterpart.

The deep-to-superficial sequence was successfully maintained in all procedures. No instances of sonographic visualization difficulty occurred, and there were no cases where air bubbles or fluid from superficial injection obscured visualization of the target to be hydrodissected, confirming the importance of the deep-first injection sequence. The pre-injection identification of the cervical sympathetic chain was consistently achieved in all patients, and caudal fluid tracking to the cervicothoracic ganglion at the C7 to T1 levels was confirmed on sagittal imaging in all cases, as illustrated in Figure 1, panel B.

Safety Outcomes

No major adverse events occurred during or following any procedure. Specifically, there were no episodes of bradycardia (heart rate below 60 bpm), hypotension (systolic blood pressure below 90 mmHg), shortness of breath, voice changes or hoarseness (suggesting recurrent laryngeal nerve involvement), dysphagia or swallowing difficulties (suggesting vagal or glossopharyngeal nerve involvement), hematoma formation, vasovagal reactions, infection, allergic reactions, intravascular injection, or nerve injury. The absence of recurrent laryngeal nerve injury is particularly notable given the proximity of this structure to the injection site, and the absence of phrenic nerve injury confirms the safety of the approach with careful needle placement.

Minor adverse events were limited to transient mild discomfort at the injection sites during needle advancement (reported in approximately 20% of procedures) and small superficial ecchymoses (noted in four patients following a single procedure each). All of these minor events resolved spontaneously without intervention within one to two days. The use of a very slow injection technique was associated with high patient satisfaction, and all patients expressed willingness to undergo the repeat procedures as scheduled.

Clinical Outcomes

All 10 patients demonstrated clinical improvement following the three-treatment series. At a minimum follow-up of 12 months post-last injection procedure, all patients sustained their clinical improvements without requiring additional interventions. Detailed clinical outcomes for these patients, including validated pain and functional scores, are reported in a separate companion manuscript.

Technical pearls and pitfalls

Essential Technical Points

Several essential technical points should be emphasized to ensure the safe and effective performance of this procedure. The deep-to-superficial injection sequence must be strictly followed, with the cervical sympathetic chain hydrodissected first, followed by the vagus nerve, as this order prevents air bubbles or fluid in the superficial planes from creating shadowing artifacts that could obscure visualization of deeper targets. Very slow injection over 12-18 min per side is critical for optimizing both patient tolerance and fluid distribution, as this rate allows tissues to gradually accommodate the volume while ensuring the injectate follows natural fascial planes to achieve complete nerve separation.

Continuous needle tip visualization is mandatory throughout the procedure, and any loss of visualization requires immediate cessation of needle advancement until the tip is confidently re-identified. Hydrodissection should always precede needle advancement, creating a halo of anechoic fluid that minimizes tissue trauma and maintains a clear visual pathway. Pre-procedural assessment of vagus nerve morphology using the reference values established by Drakonaki et al. should be routinely performed, as recognizing normal variations such as the monofascicular or oligofascicular pattern and the physiological asymmetry between right and left sides aids in accurate nerve identification and helps distinguish the vagus nerve from the smaller cervical sympathetic chain [26].

For sympathetic chain access, the needle bevel should be turned downward upon reaching the prevertebral fascia to direct injectate flow between the fascia and the longus colli muscle, as demonstrated in Figure 1, panel A [27]. Caudal tracking should be confirmed using sagittal imaging to verify that fluid extends to the C7-T1 level for adequate stellate ganglion involvement, as illustrated in Figure 1, panel B [27]. All relevant anatomy should be identified before needle insertion, with color or power Doppler used to confirm vascular structures, particularly the vertebral vessels that are critical to avoid. Without Doppler confirmation, the vertebral artery and vein may be mistaken for the C7 nerve root, potentially leading to inadvertent intravascular injection with catastrophic consequences.

Patient communication must be maintained throughout the procedure, and the very slow injection rate facilitates this by allowing ample time for feedback between syringe exchanges. Bilateral same-session treatment is enabled by the absence of local anesthetic, eliminating concern for sympathetic or vagal blockade. Finally, post-procedure monitoring should be conducted for at least 30 min to observe for any delayed adverse effects such as bradycardia, hypotension, or vasovagal reactions.

Common Pitfalls and Solutions 

Several common pitfalls may be encountered during the procedure, along with their corresponding solutions. Poor visualization of the vagus nerve can occur and is typically addressed by adjusting the depth and gain settings, using a higher-frequency transducer, and focusing identification efforts at the C6 and C7 levels, where the nerve is consistently located between the common carotid artery and the internal jugular vein. Difficulty distinguishing the vagus nerve from the sympathetic chain may be encountered, but these structures can be reliably differentiated by recognizing that the vagus nerve resides within the carotid sheath between the carotid artery and internal jugular vein, while the sympathetic chain lies posterior to the prevertebral fascia overlying the longus colli muscle, as clearly demonstrated in Figure 2, panel A.

Air bubbles obscuring the deeper view can be prevented by adhering to the deep-to-superficial injection sequence, hydrodissecting the sympathetic chain first, ensuring that all syringes and needles are meticulously primed to eliminate air, and maintaining a consistently slow injection rate throughout the procedure. The risk of intravascular injection is minimized through continuous needle tip visualization, performing negative aspiration before the initial injection, and carefully observing for any signs of vessel distension during fluid delivery. Particular attention must be paid to the vertebral vessels, as their appearance may mimic neural structures on gray-scale imaging; color Doppler or power Doppler confirmation is essential before needle advancement in this region.

Patient discomfort during the procedure can be effectively minimized by employing a very slow injection rate, ensuring that the 5% dextrose in water is warmed to approximately body temperature, performing adequate hydrodissection ahead of the needle tip to create a fluid buffer, and pausing briefly if the patient reports any discomfort. Incomplete hydrodissection may occur and can be resolved by ensuring that an adequate volume of 30 mL is delivered to each target structure and, if necessary, by redirecting the needle to address any residual perineural adhesions.

Concern regarding bilateral vagal blockade is not applicable with this technique, as no local anesthetic is used; 5% dextrose in water restores neural function rather than blocking it. Post-procedure dizziness, which is typically vasovagal in nature, can be managed by ensuring the patient is well hydrated prior to the procedure and facilitating a gradual return to an upright position upon completion.

If the injectate is not tracking caudally along the prevertebral fascia, the operator should confirm that the needle bevel is correctly oriented downward and verify that the needle tip is accurately positioned in the plane between the prevertebral fascia and the longus colli muscle. If a patient exhibits intolerance to the full 30 mL volume for a given structure, the procedure should be paused, and after a brief rest, injection can be resumed at an even slower rate. In our clinical experience, all patients ultimately tolerated the full volume using this adaptive approach.

## Discussion

The present technical report describes a novel approach to ultrasound-guided simultaneous bilateral hydrodissection of the cervical sympathetic chain, performed at the C7 vertebral level with caudal tracking to the stellate ganglion and the vagus nerves, using 5% dextrose in water without local anesthetic. The report emphasizes the critical deep-to-superficial injection sequence and the importance of very slow injection for patient tolerance. The procedure was successfully performed on 10 patients across 30 treatment sessions with no major adverse events, demonstrating its technical feasibility, safety, and excellent tolerability when the full 60 mL per side (30 mL for the sympathetic chain and 30 mL for the vagus nerve) is delivered slowly over 12-18 min per side. All patients sustained their clinical improvements at a minimum follow-up of 12 months post-last injection procedure.

Anatomical rationale

The C7 vertebral level offers distinct advantages for cervical autonomic nerve hydrodissection [29-31,36]. At this level, the absence of an anterior tubercle allows clear identification of the C7 nerve root and posterior tubercle, which serve as key landmarks for accessing the prevertebral space. The vertebral artery and vein are typically located medial to the C7 nerve root, and their identification using color Doppler or power Doppler is essential to avoid inadvertent intravascular injection, as these vessels may otherwise be mistaken for neural structures on gray-scale imaging. The longus colli muscle remains a critical landmark, with the needle positioned deep to the prevertebral fascia and superficial to the muscle to achieve optimal hydrodissection of the cervical sympathetic chain [27].

The vagus nerve at the C7 level is consistently located between and slightly posterior to the common carotid artery and internal jugular vein, appearing as a hyperechoic oval or triangular structure [37,38]. The normal morphological features described by Drakonaki et al.-including the mono- or oligo-fascicular honeycomb pattern, the asymmetry between right and left sides, and the correlation with body mass index-provide important reference standards for identifying the nerve and assessing for pathology [26]. The cervical sympathetic chain lies posterior to the carotid sheath, embedded in the prevertebral fascia overlying the longus colli muscle, appearing as a smaller hypoechoic structure [39,40]. The stellate ganglion, located more caudally at the C7 and T1 levels, can be reached by fluid tracking within the prevertebral fascia when the needle is properly positioned at C7 with bevel-down orientation and when an adequate volume (30 mL) is delivered slowly to allow caudal progression of the injectate [27].

Critical importance of deep-to-superficial injection sequence

The deep-to-superficial sequence, with the cervical sympathetic chain hydrodissected first, followed by the vagus nerve, represents a fundamental principle when performing hydrodissection of multiple targets within the same anatomical region. This sequence is not merely a matter of procedural preference but is essential for several compelling reasons that collectively determine the success, safety, and efficiency of the intervention.

First, preservation of sonographic visualization is achieved by injecting the deeper structure, which ensures that the ultrasound beam passes through undisturbed superficial tissues, thereby providing clear, unobstructed imaging of the needle tip and the target structure throughout the procedure. If the superficial structure were injected first, even microscopic air bubbles inadvertently introduced during the initial injection would create hyperechoic artifacts that cast acoustic shadows, thereby obscuring visualization of the deeper target [32]. This degradation of image quality can persist throughout the remainder of the procedure and may compromise the operator's ability to safely guide the needle to the intended location.

Second, avoidance of anatomical distortion is accomplished because fluid accumulation in the superficial plane, namely the carotid sheath, compresses potential spaces and alters the normal relationship between fascial planes. This distortion makes subsequent access to the deeper prevertebral space significantly more difficult and increases the risk of unintended needle placement. When the deeper space is accessed first, the tissue planes remain in their native configuration, providing reliable anatomical guidance.

Third, landmark identification is maintained because the carotid artery, internal jugular vein, and vagus nerve serve as important sonographic landmarks for accessing the deeper sympathetic chain. Injecting the vagus nerve first would inevitably displace these structures and alter their spatial relationships, thereby compromising their utility as reliable guides to the deeper target. Maintaining these landmarks in their undisturbed state facilitates accurate and efficient needle navigation.

Fourth, efficiency is enhanced because the deep-to-superficial sequence allows both targets to be addressed through a single skin entry and a continuous needle trajectory, requiring only slight withdrawal and redirection between targets. This approach minimizes tissue trauma, reduces procedure time, and eliminates the need for multiple skin punctures.

Finally, safety is promoted because clear, uninterrupted visualization throughout the procedure substantially reduces the risk of inadvertent vascular puncture or nerve trauma. The operator can maintain continuous awareness of the needle tip position relative to all critical structures, thereby avoiding complications that might arise from working in distorted or poorly visualized anatomy.

Critical importance of slow injection rate

The very slow injection rate of 12-18 min per side is equally critical to the success of this procedure and represents a deliberate departure from conventional injection techniques. This technical factor has several important implications that extend far beyond mere patient comfort.

First, patient comfort is markedly enhanced by slow injection. Rapid distension of tissue planes can cause acute pain and anxiety, which may lead to involuntary patient movement, procedural interruption, or even abandonment of the procedure. The slow rate allows the tissues to accommodate the expanding fluid volume gradually, and patients consistently report minimal to no discomfort during the procedure. This positive patient experience is essential for maintaining trust and cooperation, particularly when multiple treatment sessions are required.

Second, optimal fluid distribution is achieved with slow injection. The hydrodissection technique fundamentally relies on fluid gently separating tissue planes along natural fascial boundaries rather than forcibly disrupting them. Rapid injection may create turbulent flow and uneven distribution of the injectate, potentially reducing the efficacy of hydrodissection and leaving residual adhesions intact. Slow, steady injection allows the fluid to follow the path of least resistance along established fascial planes, achieving more complete and uniform separation of the target nerve from surrounding tissues.

Third, the ability to deliver the full 60 mL per side depends critically on slow injection. In our study, all patients tolerated the complete volume with the slow injection technique. In the few instances where mild discomfort prompted a brief pause, resuming at an even slower rate allowed completion of the full volume without exception. No patient required reduction of the target volume, underscoring the effectiveness of this approach in maximizing therapeutic delivery.

Fourth, slow injection facilitates continuous communication with the patient, allowing real-time feedback and early detection of any potential issues. The extended procedure time provides ample opportunity for the operator to assess the patient's response, answer questions, and address any concerns as they arise. This ongoing dialogue enhances patient safety and satisfaction while allowing the operator to make minute adjustments to technique based on immediate feedback.

Finally, slow injection may enhance caudal tracking of fluid to the stellate ganglion. The 30 mL volume for sympathetic chain hydrodissection, when delivered slowly, has sufficient time to track along the prevertebral fascia to reach the C7 and T1 levels, as confirmed on sagittal imaging in all our cases. Rapid injection might result in fluid preferentially following a path of lesser resistance locally rather than extending distally along the fascial plane, thereby failing to reach the stellate ganglion region.

Advantages of 5% dextrose in water without local anesthetic

The use of 5% dextrose in water (D5W) without local anesthetic is fundamental to this technique and distinguishes it from traditional interventional approaches to the cervical autonomic nerves. This choice of injectate offers multiple distinct advantages that collectively enable the unique capabilities of this procedure.

Regarding safety for bilateral treatment, local anesthetics would pose an unacceptable risk of bilateral sympathetic and vagal blockade with potentially serious consequences, including bradycardia, hypotension, and respiratory compromise. D5W has no nerve-blocking properties whatsoever and appears to restore normal neural function rather than suppressing it, as evidenced by the absence of adverse effects in numerous studies of peripheral nerve hydrodissection [18-20]. This safety profile is what makes same-session bilateral treatment feasible and practical.

Regarding mechanism of action, D5W provides mechanical release of nerves from fascial restrictions through hydrodissection while simultaneously exerting beneficial metabolic effects on inflamed neural tissue. Laboratory investigations have demonstrated that glucose treatment reduces reactive oxygen species production and apoptosis in inflamed neural cells in vitro, suggesting a direct therapeutic effect beyond simple mechanical release [21,22]. This contrasts sharply with local anesthetics that temporarily block neural transmission without addressing the underlying pathophysiology of nerve dysfunction and inflammation.

Regarding absence of neurotoxicity, D5W is iso-osmolar and non-neurotoxic, unlike some local anesthetics that can be neurotoxic at clinical concentrations, particularly when injected intrafascicularly or at high concentrations [41,42]. The safety margin with D5W is exceptionally wide, allowing the administration of larger volumes without concern for chemical neurotoxicity.

Regarding patient tolerance, D5W injections are well-tolerated with minimal discomfort, particularly when hydrodissection is performed slowly ahead of the needle and the solution is warmed to approximately body temperature. Patients typically report a sensation of pressure rather than pain, and the absence of the burning sensation sometimes associated with local anesthetic injection enhances the overall patient experience.

Volume considerations

The selection of 30 mL for sympathetic chain hydrodissection and 30 mL for vagus nerve hydrodissection, totaling 60 mL per side, is based on several interrelated considerations derived from anatomical principles and clinical experience.

First, the cervical sympathetic chain and stellate ganglion region require adequate volume to track caudally along the prevertebral fascia from the C7 level to the cervicothoracic junction. The 30 mL volume for sympathetic hydrodissection, when delivered slowly with the bevel oriented downward, consistently achieved this caudal tracking in our series, as confirmed on sagittal imaging in all cases. Smaller volumes might remain localized and fail to reach the stellate ganglion, thereby limiting the therapeutic effect.

Second, the carotid sheath is a capacious potential space that can accommodate significant volume without causing undue pressure on adjacent structures. The vagus nerve lies within this sheath, and 30 mL of injectate reliably achieves 360° separation of the nerve from the surrounding carotid artery, internal jugular vein, and fascial attachments. This complete circumferential release is essential for optimal therapeutic effect, as residual adhesions could perpetuate nerve dysfunction.

Third, the total volume of 60 mL per side is well within the safety limits for subcutaneous and fascial injection in the cervical region, particularly given the slow injection rate and the absence of local anesthetic. The cervical tissues are surprisingly accommodating to volume when injection is performed slowly, and we observed no evidence of excessive pressure, tissue ischemia, or other volume-related complications.

Finally, in the rare event that a patient cannot tolerate the full volume, the slow injection technique allows for pausing and resuming, and all patients in our series ultimately tolerated the complete volume. This adaptability ensures that therapeutic goals are not compromised while maintaining patient comfort and safety.

Comparison to traditional sympathetic blocks

Traditional stellate ganglion blocks have long utilized local anesthetic solutions injected as a therapeutic block for various conditions, including complex regional pain syndrome (types I and II), post-herpetic neuralgia, chronic pain syndromes affecting the head and neck, vascular insufficiency conditions, hyperhidrosis, and more recently, post-traumatic stress disorder [7-9,15-17]. These procedures are typically performed using small volumes of local anesthetic, ranging from 5 to 10 mL, and are conventionally executed at the C6 vertebral level, where the prominent anterior tubercle (Chassaignac tubercle) serves as a reliable bony landmark. The advent of ultrasound guidance has significantly enhanced the safety and precision of these blocks, allowing direct visualization of soft tissues, preventing complications such as intravascular injection or recurrent laryngeal nerve block, and enabling accurate subfascial deposition of the anesthetic agent under real-time imaging [43,44].

Despite these advancements, local anesthetic sympathetic blocks possess inherent limitations that constrain their therapeutic utility. Their effects are temporary, typically lasting only hours to days, which necessitates repeated interventions for sustained symptom relief. This transient nature can lead to a cycle of frequent clinic visits, cumulative radiation exposure if fluoroscopy is used, and significant healthcare costs. The procedure carries inherent risks, including hematoma formation from vascular puncture, unintended blockade of the recurrent laryngeal nerve resulting in transient hoarseness and dysphagia, and the potential for intravascular injection with systemic local anesthetic toxicity. Perhaps most significantly, bilateral stellate ganglion blocks are contraindicated due to the unacceptable risk of bilateral sympathetic or vagal blockade, which could precipitate severe bradycardia, hypotension, or respiratory compromise requiring emergency intervention. Furthermore, local anesthetics provide purely symptomatic relief through temporary nerve blockade and offer no tissue-healing properties or anti-inflammatory effects that address the underlying pathophysiology of the target condition.

In contrast, the hydrodissection approach utilizing 5% dextrose in water offers several distinct and compelling advantages. It provides mechanical release of perineural fascial restrictions through hydraulic force while simultaneously exerting beneficial metabolic and anti-inflammatory effects on inflamed neural tissue, including the reduction of reactive oxygen species production and inhibition of apoptotic pathways [21,22]. The therapeutic benefits appear substantially more durable, with patients in our series achieving sustained improvement following just three monthly treatments. The absence of local anesthetic eliminates concerns about bilateral autonomic blockade, thereby enabling safe same-session bilateral treatment, a capability not possible with traditional approaches. This technique uniquely addresses both branches of the autonomic nervous system simultaneously - sympathetic overactivity via cervical sympathetic chain and stellate ganglion hydrodissection, and parasympathetic insufficiency via vagus nerve hydrodissection. Moreover, the C7-level approach, with careful Doppler identification and avoidance of the vertebral vessels, allows more direct access to the stellate ganglion region than the traditional C6 approach. The substantially larger volume of 60 mL per side facilitates more extensive hydrodissection along fascial planes, potentially addressing more extensive perineural adhesions than traditional small-volume blocks can achieve.

Comparison to other vagus nerve approaches

Invasive vagus nerve stimulation represents an established therapeutic modality for certain forms of epilepsy, depression, and more recently, migraine and cluster headaches. This approach requires surgical implantation of a pulse generator and electrode, typically in the left cervical region, and carries inherent risks including surgical site infection, hematoma formation, vocal cord paralysis from recurrent laryngeal nerve injury, and device-related complications such as lead fracture or migration [45,46]. The procedure is expensive, requires specialized surgical expertise, and is not widely accessible to all patient populations. Furthermore, device implantation is typically reserved for patients with severe, treatment-refractory conditions due to its invasive nature.

Non-invasive transcutaneous vagus nerve stimulation has emerged as a more accessible alternative, delivering electrical stimulation through the skin via devices applied to the cervical region or the auricular branch of the vagus nerve in the ear. While this approach avoids surgical risks, it requires daily or twice-daily application for sustained benefit, and patient adherence to such regimens can be challenging. The efficacy and durability of transcutaneous stimulation continue to require further confirmation through rigorous clinical trials, and the optimal stimulation parameters remain an area of active investigation [47,48]. Importantly, current recommendations favor left-sided stimulation due to concerns that right-sided or bilateral stimulation might precipitate bradycardia or other cardiac arrhythmias [49].

The hydrodissection approach described in this report offers a compelling alternative with several distinct advantages. It is minimally invasive, requiring only a small-gauge needle and no surgical implantation. It enables bilateral treatment in a single session without any risk of bradycardia, as 5% dextrose in water has no nerve-blocking properties and appears to restore normal neural function rather than suppressing it. The procedure provides both mechanical and biological therapeutic effects, addressing fascial restrictions while promoting neural recovery through metabolic mechanisms. Durable benefits are achieved after only three monthly treatments, in contrast to the daily regimens required for transcutaneous stimulation. Most significantly, this approach simultaneously addresses both branches of the autonomic nervous system - the sympathetic chain and the vagus nerve-offering a comprehensive therapeutic strategy for conditions characterized by autonomic dysregulation.

Safety considerations

The proximity of numerous critical structures within the confined anatomical space of the neck necessitates meticulous technique and thorough anatomical knowledge to ensure patient safety. Several key safety considerations warrant particular emphasis.

Regarding vascular injury, the common carotid artery and internal jugular vein lie in immediate proximity to both target nerves within the carotid sheath. The risk of intravascular penetration is minimized through continuous needle tip visualization throughout the procedure and by maintaining the fundamental principle of hydrodissection ahead of the needle, whereby fluid is injected to create a safe pathway before the needle advances into any new tissue plane. During vagus nerve hydrodissection, particular vigilance is required as the needle is intentionally directed into the carotid sheath, placing these vessels at direct risk. Inadvertent puncture of the common carotid artery may result in hematoma formation, vessel dissection, or, in rare cases, thromboembolic complications, while puncture of the internal jugular vein carries risks of hematoma, venous thrombosis, or air embolism. Negative aspiration before initiating injection and careful observation for any signs of vessel distension during fluid delivery are essential additional safety measures.

Regarding the vertebral artery and vein, these vital structures are typically located medial to the C7 nerve root at the level of the C7 posterior tubercle. The routine use of color or power Doppler is absolutely essential to distinguish these vessels from adjacent neural structures, as without Doppler signal, the vertebral artery and vein may be mistaken for the C7 nerve root, potentially leading to inadvertent intravascular injection with catastrophic consequences including hematoma, seizure, or stroke. This distinction is particularly critical when working at the C7 level, where the absence of an anterior tubercle alters the usual landmark relationships.

Regarding the cervical nerve roots, particularly C5, C6, and C7, these critical neural structures must be avoided during needle advancement. The C7 nerve root lies between the vertebral artery medially and the posterior tubercle of C7 laterally, while the C5 and C6 nerve roots lie between their respective anterior and posterior tubercles at higher levels. Inadvertent needle contact or injection into a nerve root may precipitate acute radicular pain, paresthesias, or, in rare cases, persistent nerve injury. Careful continuous visualization of the needle tip throughout the procedure, combined with the technique of hydrodissection ahead of the needle, minimizes the risk of unintended neural contact.

Regarding the phrenic nerve, this structure originates from the C3, C4, and C5 nerve roots and courses obliquely across the anterior surface of the scalenus anterior muscle, positioned directly in the path of needle advancement toward the prevertebral fascia. Injury to the phrenic nerve may result in diaphragmatic dysfunction or hemidiaphragmatic paralysis, which can present clinically as dyspnea, orthopnea, or reduced exercise tolerance. The risk of phrenic nerve injury is minimized by maintaining the needle in a plane deep to the prevertebral fascia, using hydrodissection to gently separate tissues ahead of the needle.

Regarding the recurrent laryngeal nerve, this important branch of the vagus nerve courses inferiorly in the tracheoesophageal groove and is vulnerable to injury if the needle is advanced too medially. Injury to this nerve may result in temporary or persistent hoarseness due to ipsilateral vocal cord paresis or paralysis. Maintaining the needle lateral to the carotid sheath and avoiding medial deviation beyond the sheath boundaries are essential preventive measures.

Regarding the esophagus, this structure is located medially on the left side of the neck and may be encountered with excessive medial needle deviation, particularly during left-sided procedures. Careful attention to needle orientation and trajectory helps avoid this complication.

Limitations

The present technical report has several important limitations that should be acknowledged. The procedure requires advanced ultrasound-guided interventional skills and a comprehensive, nuanced understanding of cervical anatomy, including the distinct anatomical features of the C7 level and the critical importance of Doppler assessment for identifying vertebral vessels. The learning curve is significant, and practitioners should have extensive experience with peripheral nerve hydrodissection in more superficial and less anatomically complex regions before attempting cervical autonomic hydrodissection.

Several technical parameters remain to be optimized through further research. The optimal volume of 5% dextrose in water, the ideal number of treatment sessions, and the most effective interval between sessions have yet to be definitively established. While we observed excellent patient tolerance with 60 mL per side delivered slowly, individual patient factors such as body habitus, tissue compliance, and pain threshold may influence the optimal volume for any given individual.

The relatively small sample size of 10 patients limits the generalizability of our findings, and the absence of a control group precludes definitive conclusions about therapeutic efficacy. It is important to emphasize that the primary purpose of this report is to establish technical feasibility and procedural safety, not to demonstrate clinical efficacy. The latter is appropriately addressed in our companion clinical outcomes manuscript, which provides a detailed analysis of validated pain and functional outcome measures.

The very slow injection technique, while critical for patient tolerance and optimal fluid distribution, requires considerable operator patience and may not be feasible in all practice settings due to time constraints or high-volume clinical demands. However, the total procedure time of 24-36 min for bilateral treatment is reasonable given the potential therapeutic benefits and compares favorably to the cumulative time required for multiple return visits for traditional sympathetic blocks.

Future directions

Several avenues for future investigation warrant exploration to further refine and validate this novel technique. Further research should address optimal treatment protocols, including systematic evaluation of different injectate volumes, number of treatment sessions, and intervals between sessions to establish evidence-based guidelines for clinical practice. Long-term durability beyond the six-month follow-up period reported here requires continued investigation to determine whether maintenance treatments are necessary and, if so, at what intervals.

Comparative studies evaluating the relative merits of the C6 versus C7 approach for sympathetic chain hydrodissection would help refine technique selection and provide guidance for operators regarding which approach may be preferable in specific clinical scenarios or for particular patient populations. The applicability of this technique to other conditions characterized by autonomic dysfunction, such as postural orthostatic tachycardia syndrome (POTS), myalgic encephalomyelitis or chronic fatigue syndrome (ME/CFS), and fibromyalgia without prior COVID-19 infection, should be systematically explored.

Mechanistic studies utilizing comprehensive autonomic function testing, heart rate variability analysis, and serial measurement of inflammatory biomarkers would enhance our understanding of the therapeutic mechanisms underlying this intervention. Such investigations could help identify which patients are most likely to benefit and provide objective correlates of clinical improvement. Finally, randomized controlled trials comparing this hydrodissection approach to sham procedures, active controls such as traditional sympathetic blocks, or standard-of-care treatments are warranted to definitively establish the efficacy and cost-effectiveness of this novel technique. Investigation of the optimal injection sequence in other multi-target regional hydrodissection procedures would also provide valuable guidance for the broader field of interventional pain management.

## Conclusions

Ultrasound-guided bilateral simultaneous hydrodissection of the cervical sympathetic chain, with caudal tracking to the cervicothoracic ganglion, and the vagus nerves using 5% dextrose in water without local anesthetic, is technically feasible and safe. The C7 level offers distinct anatomical advantages, and Doppler confirmation of the vertebral vessels is essential to prevent inadvertent intravascular injection. The deep-to-superficial injection sequence (sympathetic chain first, vagus nerve second) is critical for maintaining optimal sonographic visualization, and very slow injection over 12-18 min per side enables delivery of the full 60 mL volume with excellent patient tolerance. In our series of 10 patients across 30 treatment sessions, no major adverse events occurred, and all patients tolerated the full volume. While preliminary clinical improvements were observed, detailed outcomes are reported in a separate companion manuscript. Larger, prospective, controlled studies are warranted to establish definitive efficacy.
